# Tanshinone IIA Pretreatment Protects H9c2 Cells against Anoxia/Reoxygenation Injury: Involvement of the Translocation of Bcl-2 to Mitochondria Mediated by 14-3-3*η*

**DOI:** 10.1155/2018/3583921

**Published:** 2018-06-28

**Authors:** Zeyu Zhang, Huan He, Yang Qiao, Jiyi Huang, Zelong Wu, Ping Xu, Dong Yin, Ming He

**Affiliations:** ^1^Jiangxi Provincial Institute of Hypertension, The First Affiliated Hospital of Nanchang University, Nanchang 330006, China; ^2^Jiangxi Provincial Key Laboratory of Basic Pharmacology, Nanchang University School of Pharmaceutical Science, Nanchang 330006, China; ^3^Jiangxi Provincial Key Laboratory of Molecular Medicine, The Second Affiliated Hospital of Nanchang University, Nanchang 330006, China

## Abstract

Tanshinone IIA is an important component that is isolated from danshen (*Salvia miltiorrhiza*), which is known to be beneficial for cardiovascular health. In this study, we determined the effects of Tanshinone IIA and its underlying mechanisms of action in an anoxia/reoxygenation (A/R) cell line model. Prior to inducing A/R injury, rat cardiomyocyte-derived cell line H9c2 was stimulated with 8 *μ*M of Tanshinone IIA for 48 hours. When compared with the A/R group, the Tanshinone IIA treatment significantly increased cell viability and decreased lactate dehydrogenase activity. Tanshinone IIA upregulated 14-3-3*η* expression and facilitated Bcl-2 translocation to the mitochondrial outer membrane, which bound with voltage-dependent anion channel 1. In addition, pretreatment with Tanshinone IIA reduced the generation of reactive oxygen species and cytochrome c release, inactivated caspase-3, prevented mitochondrial permeability transition pore opening, and reduced the percentage of apoptotic cells. Moreover, treatment with Tanshinone IIA reduced the level of malondialdehyde, thereby increasing the activity of superoxide dismutase and glutathione peroxidase. Silencing the expression of 14-3-3*η* by adenovirus blocked the above-mentioned results. These novel findings showed that pretreatment with Tanshinone IIA alleviated H9c2 cell damage against A/R injury and was associated with upregulation of 14-3-3*η*, thereby facilitating Bcl-2 translocation to the mitochondrial outer membrane and preventing mitochondrial permeability transition pore opening, decreasing cytochrome c release, preventing caspase-3 activation, and restraining apoptosis.

## 1. Introduction

Danshen (*Salvia miltiorrhiza*) is a traditional Chinese medicine that is used worldwide to improve blood flow and blood stasis [1]. Tanshinone IIA (TSN) is a natural active ingredient that is derived from danshen. Previous studies have shown that TSN plays a biological role in antioxidant and anti-inflammatory activities *in vivo* [2, 3]. Recently, TSN has been demonstrated to provide significant protection against ischemia-reperfusion injury in various tissues [4–6]. Interestingly, in rat *in vivo* study, it was shown that infarct size was significantly reduced in a myocardial ischemia model that was pretreated with TSN for one week [7]. However, this study only involved pathological results including biomarkers of oxidative stress and apoptosis; the mechanism of action involving this phenomenon was not explored.

Reperfusion injury plays an important role in death caused by ischemic cardiovascular diseases [8]. Ischemic preconditioning (IPC) and pharmacological preconditioning (PPC) are the most common strategies to prevent lethal reperfusion injury [8, 9]. However, clinical outcomes in IPC and PPC were marginally successful [9]. Recently, Abdukeyum et al. [10] proposed that nutrition preconditioning (NPC) could be considered as a novel beneficial approach to alleviate lethal reperfusion injury, which was confirmed by our previous studies in which this was further defined [11]. When compared with IPC, NPC has a noninferiority effect and involves more feasible implementation methods [11, 12].

The Bcl-2 family of proteins plays an important role in mitochondria-mediated apoptosis [13]. Previous studies have shown that Bcl-2 could inhibit mitochondrial permeability transition pore (mPTP) opening through directly binding with the voltage-dependent anion channel 1 (VDAC-1) which is considered the mPTP's gate of ion exchange [11, 13]. Opening of the mPTP will lead to outflow of proapoptosis factors, such as outflow of cytochrome C (cyt c) and activation of caspase cascades [14]. The mechanism of how Bcl-2 binding with VDAC-1 occurs has not been described. Our previous studies demonstrated that 14-3-3*η* facilitated the translocation of proteins into mitochondria (unpublished results). Therefore, we hypothesized that 14-3-3*η* is a feasible target of TSN-mediated cardioprotective effects and that the proposed mechanism of action involves 14-3-3*η* that supports Bcl-2 to translocate into the mitochondria where it binds with VDAC-1. To confirm this hypothesis, we used an anoxia/reoxygenation (A/R) model in H9c2 cells to simulate reperfusion injury and investigated the relation between TSN and 14-3-3*η* by RNAi technology. Moreover, we also evaluated the potential interaction and function among 14-3-3*η*, Bcl-2, and VDAC-1 in this model.

## 2. Materials and Methods

### 2.1. Cell Culture and A/R Injury Model

Cell culture components were purchased from Life Technologies (Paisley, Scotland). AD14-3-3*η* RNAi (5′-AAGCTTCTGAG GCAGCGTATA-3′), AD-scrRNAi (5′-TTC-TCCGAACGTGTCACGT-3′) and rat cardiomyocyte-derived cell line H9c2 were purchased from Shanghai Genechem Co., Ltd. (Shanghai, China). TSN was purchased from the Chinese Institute of Pharmaceutical Biological Products Analysis (Beijing, China). H9c2 cells were cultured in high-glucose Dulbecco's modified Eagle medium (DMEM), containing 10% fetal bovine serum (FBS). Cells were cultured at 37°C in a 95% O_2_ and 5% CO_2_ incubator. A/R treatment and anoxia preconditioning (APC) methods were performed as previously described [15, 16]. In brief, cells were treated with different concentrations of TSN (2, 8, and 32 *μ*M) at different times prior to the experiment (12, 24, 36, and 48 hours). For investigating the cardioprotection of TSN, cells were divided into seven groups: (1) control group: cells were incubated with high-glucose DMEM containing 10% FBS without adenovirus-silencing 14-3-3*η* and A/R induction; (2) A/R group: H9c2 cells were exposed to anoxia by incubation with anoxia medium for 3 h and were reoxygenated for 2 h with reoxygenation medium as previously described [15]; (3) TSN + A/R group: cells treated with 8 *μ*M TSN for 48 hours before induction of A/R; (4) TSN + AD14-3-3*η* RNAi + A/R group: cells were treated with 8 *μ*M TSN for 48 hours and infected with AD14-3-3*η*RNAi for 48 hours before induction of A/R; (5) AD14-3-3*η*RNAi + A/R group: cells were infected with AD14-3-3*η*RNAi for 48 hours prior to induction of A/R; (6) AD-scrRNAi + A/R group: cells were infected with AD-scrRNAi for 48 hours prior to induction of A/R; and (7) APC + A/R group: cells were treated with APC and incubated with high-glucose DMEM containing 10% FBS for 24 hours prior to induction of A/R.

### 2.2. Lactate Dehydrogenase (LDH) Activity and Cell Viability Assay

LDH is an intracellular enzyme of cardiomyocytes, which is released into the culture medium during cellular damage. Therefore, after treatment, supernatant was collected and the activity of LDH was determined by a microplate reader (Bio-Rad 680, Hercules, CA, USA). Cell viability was assessed by methyl thiazolyl tetrazolium (MTT) assay. In brief, cells were plated in 96-well plates at a density of 1 × 10^5^ cells/well. After treatment, as described in Section 2.1, cells were incubated with MTT (0.5 mg/ml) in phosphate-buffered saline (PBS) for 4 hours at 37°C in the dark. Then, the medium was replaced with 150 *μ*l dimethyl sulfoxide (DMSO) and analyzed by a microplate reader.

### 2.3. Determination of Lipid Peroxidation and Antioxidant Enzyme Activities

Malondialdehyde (MDA), superoxide dismutase (SOD), and glutathione peroxidase (GSH-Px) are the most important biomarkers for estimating oxidative stress. After treatment, the supernatant of cellular lysis was collected and measured according to the manufacturer's guidelines. All collected supernatants were measured using a microplate reader (Bio-Rad 680, Hercules, CA, USA). The kits for measuring MDA, SOD, and GSH-Px were purchased from Nanjing Jiancheng Bioengineering Institute (Nanjing, China).

### 2.4. Isolation of Mitochondrial and Cytosolic Fractions

At termination of the A/R model, cells were collected and washed twice with ice-cold PBS. Then, cells were resuspended in 1x cytosol extraction buffer and homogenized on ice. The homogenate was transferred to a new tube and vortexed for 15 sec. Then, the homogenization buffer was placed on ice for 15 min and centrifuged at 3000 × rpm/10 min at 4°C, discarding the pellet. Next, the supernatant was transferred into a new tube and centrifuged at 12000 rpm for 30 min at 4°C to precipitate mitochondria. The supernatant, containing cytoplasmic proteins, was frozen and stored for Western blot analysis. To purify mitochondria, the pellet was dissolved with 1x cytosol extraction buffer and centrifuged at 12000 rpm for 30 min at 4°C. The pellet was resuspended with mitochondrial lysis buffer and placed on ice for 30 min. Then, the mixture was vortexed for 30 sec and centrifuged at 13000 rpm for 10 min at 4°C. The pellet, containing mitochondrial proteins, was evaluated by Western blot analysis.

### 2.5. Measurement of Mitochondrial Permeability Transition Pore (mPTP) Opening

Opening of the mPTP was performed as previously described [17]. In brief, mitochondria were resuspended in swelling buffer (5 mmol/l KH_2_PO_4_, 120 mmol/l KCl, 10 mmol/l [pH 7.4], and 20 mmol/l MOPS) to 0.25 mg/ml and added to 200 *μ*mol/l CaCl_2_ to induce opening of the pore. Then, absorbance was determined by a spectrophotometer at 520 nm every 30 sec for a total of 30 min. The negative control for this assay was 30 nmol/l cyclosporin A.

### 2.6. Coimmunoprecipitation Assay

Induced by A/R injury, cardiomyocytes were placed on ice and homogenized in radioimmunoprecipitation assay (RIPA) lysis buffer (Solarbio, Beijing, China) with a protease inhibitor. The homogenates were centrifuged at 10000 ×g for 15 min at 4°C, and the sediment was discarded. Protein concentration in the supernatant was measured by bicinchoninic acid (BCA) assay. 100 *μ*g protein was added to anti-VDAC-1 (Santa Cruz, CA, USA), anti-Bcl-2 (Abcam, Cambridge, MA, USA), and anti-14-3-3*η* (Abcam, Cambridge, MA, USA) at 33 *μ*g protein per 1 *μ*g of antibody and incubated overnight at 4°C. Protein A/G plus beads (Santa Cruz, CA, USA) were washed with cold PBS prior to use and added to the mixture. Mixtures were incubated at 4°C for 4 hours and centrifuged at 600 ×g for 3 min at 4°C. Pellets were washed three times with precold PBS. The immunoprecipitation complex was mixed with 1x sodium dodecyl sulfate-polyacrylamide gel electrophoresis (SDS/PAGE) gel sample buffer and boiled for 5 min; then, proteins were analyzed by Western blot analysis.

### 2.7. Western Blotting Analysis

Proteins were extracted using RIPA lysis buffer, containing protease inhibitors. The extractive was centrifuged at 12000 ×g for 25 min at 4°C to remove insoluble pellets. A total of 30 *μ*g of proteins were boiled in 1x SDS-PAGE sample buffer for 5 min and separated on a 10% SDS-PAGE gel. Then, proteins were transferred to a polyvinylidene fluoride (PVDF) membrane, which was activated by methanol and blocked with 10% skim milk in Tris-buffered saline, containing 0.1% Tween-20 (TBST) for 2 hours at 20°C. Then, membranes were incubated with primary antibodies directed against VDAC-1, Bcl-2, 14-3-3*η*, *β*-actin (ZSGB-Bio, China), or cyt C (Santa Cruz, CA, USA) at 4°C overnight, washed for 15 min for 3 times, and incubated with a corresponding horseradish peroxidase- (HRP-) conjugated secondary antibody for 2 hours at 20°C. Then, membranes were incubated with enhanced chemiluminescence (ECL) reagent for 1 min and proteins were visualized by a GDS-8000 UVP photo scanner and Image Lab software (Bio-Rad, Hercules, CA, USA) or a preflashed X-ray film (Fujifilm, Tokyo, Japan). The intensities of the protein bands were assessed by Image Lab software (Bio-Rad, Hercules, CA, USA).

### 2.8. Fluorescence and Confocal Microscopy

To assess for VDAC-1, Bcl-2 and 14-3-3*η* colocalization, cardiomyocytes were placed on a confocal-only plate (Nest, Wuxi, China). After treatment, as described in Section 2.1, cells were fixed with 4% paraformaldehyde, permeabilized with 0.5% Triton X-100 (diluted in PBS) on ice, and blocked in 5% bovine serum albumin (BSA) buffer (diluted in PBS). Then, cells were cultured overnight in the presence of goat-anti-VDAC-1 (1 : 50), rabbit-anti-Bcl-2 (1 : 50), and mouse-anti-14-3-3*η* (1 : 200) at 4°C. Subsequently, cells were cultured in an incubator at 37°C, washed three times with PBS, and incubated with orange donkey anti-goat IgG, red donkey anti-rabbit IgG, and green donkey anti-mouse IgG (Abbkine, Redlands, CA, USA). Nuclei were stained for 3 min with 4′,6-diamidino-2-phenylindole (DAPI) in the dark at 20°C. Then, cells were imaged using confocal microscopy (ZEISS LSM 700, Germany). Colocalization of VDAC-1, Bcl-2, and 14-3-3*η* was analyzed by ZEN 2.1 SP1 software (ZEISS, Shanghai, China). For ease in presenting the data, we decided to turn the color green into blue, blue into yellow, and orange into green. For each group, at least 6 cells were randomly assessed and all experiments were performed three times.

### 2.9. Caspase-3 Activity Assay

Caspase-3 activity was determined by the caspase-3 activity assay kit (Beyotime, Shanghai, China). In brief, a standard curve was created using different concentrations of the standard *p*-nitroanilide (pNA). Cells were collected and washed with ice-cold PBS. Then, cells were incubated for 15 min on ice and centrifuged at 16000 ×g for 15 min, and the supernatant was transferred to a new tube. The supernatant was incubated with mix reagent (detection buffer and Ac-DEVE-pNA) at 37°C for 2 hours and analyzed using a microplate reader.

### 2.10. Flow Cytometry Analysis of Intracellular Reactive Oxygen Species (ROS) and Cardiomyocyte Apoptosis

Prior to analysis of ROS by flow cytometry, cells were collected after treatment which was described in Section 2.1 and incubated with a fluorescent probe 10 *μ*M 2′,7′-dichlorofluorescein diacetate (DCFH-DA, KeyGen, Nanjing, China). Then, samples were centrifuged at 600 ×g for 5 min, washed three times with cold PBS, and analyzed by flow cytometry (excitation: 488 nm; emission: 525 nm). Cardiomyocyte apoptosis was determined by Annexin V/PI kit (BestBio, Shanghai, China). In brief, cells were washed with ice-cold PBS and collected. Then, cells were resuspended in 1x Annexin V Binding Buffer (250 *μ*l). Cells were mixed with 5 *μ*l Annexin V-FITC dye and incubated at 4°C for 15 min in the dark. Cells were mixed with 10 *μ*l propidium iodide dye, incubated at 4°C for 5 min in the dark, and analyzed by flow cytometry.

### 2.11. Statistical Analysis

Data are presented as the mean ± SEM. Differences between groups were analyzed using one-way ANOVA followed by least-significant difference post hoc testing for individual differences using SPSS 11. *P* < 0.05 was considered statistically significant.

## 3. Results

### 3.1. Effects of TSN Pretreatment on LDH Activity, Cell Viability, and 14-3-3*η* Expression in H9c2 Cells Subjected to A/R

**Figure 1:**
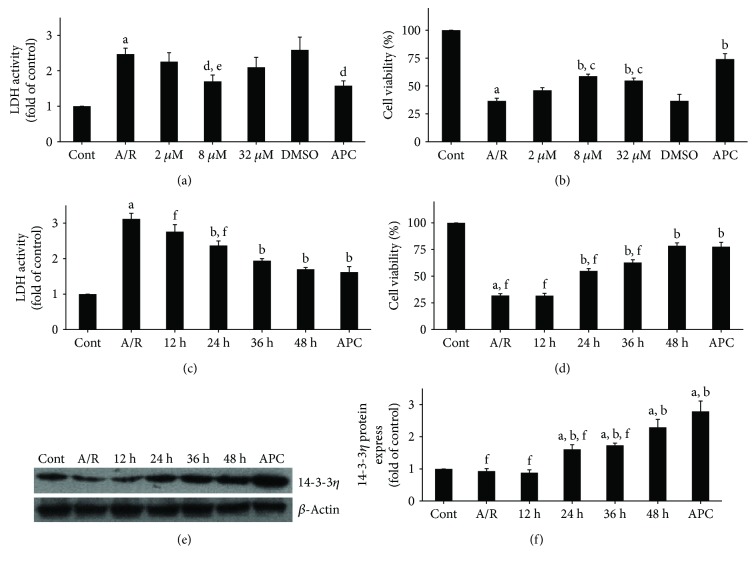
Effects of Tanshinone IIA (TSN) preconditioning on lactate dehydrogenase activity, cell viability, and 14-3-3*η* expression in H9c2 cells subjected to anoxia/reoxygenation (A/R). (a, b) Cells were treated in a dose-dependent manner with 2 *μ*M to 32 *μ*M for 24 hours prior to induction of A/R. (c, d, e, f) Cells were treated in a time-dependent manner from 12 hours to 48 hours prior to A/R. (e, f) The time-dependent effect of TSN preconditioning on 14-3-3*η* expression. Control group represents cells without any treatment. *β*-Actin served as a loading control. Values are presented as the mean ± SEM for five individual experiments. (A) *P* < 0.01 versus control group; (B) *P* < 0.01 versus A/R group; (C) *P* < 0.01 versus 1% DMSO group; (D) *P* < 0.05 versus A/R group; (E) *P* < 0.05 versus 1% DMSO group; (F) *P* < 0.01 versus 8 *μ*M TSN + A/R (48 hours) group.

LDH often serves as a marker to predict cardiomyocyte condition. MTT assays were performed to assess cardiomyocyte viability. To evaluate the effect of TSN on cardiomyocytes, we tested different concentrations of TSN on H9c2 cells that were subjected to A/R injury. Compared with A/R, treatment with 8 *μ*M TSN and APC significantly reduced LDH activities (*P* < 0.01) and improved cell viability (*P* < 0.01; Figures 1(a) and 1(b)). Moreover, we found a time-dependent protective effect of TSN in H9c2 cells subjected to A/R injury. As shown in Figures 1(c) and 1(d), LDH activities showed a time-dependent decrease, whereas cell viability showed a time-dependent increase. Interestingly, we found a time-dependent upregulation in 14-3-3*η* expression (Figures 1(e) and 1(f)). The expression of 14-3-3*η* was also upregulated in the APC group (*P* < 0.01 versus the control group). These results indicated that the protective effect of pretreatment with 8 *μ*M TSN over 48 hours was similar to that of the APC group. These data confirmed the protective effect of TSN and suggested that its effect involves the expression of 14-3-3*η*.

### 3.2. 14-3-3*η* Low Expression Abolished the Protective Effect of TSN Preconditioning on LDH Activity and Cell Viability in H9c2 Cells Subjected to A/R Injury

**Figure 2:**
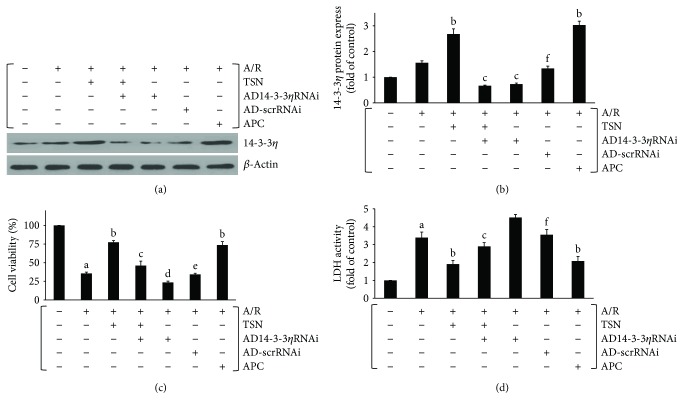
Low expression of 14-3-3*η* abolished the protective effect of Tanshinone IIA (TSN) preconditioning on lactate dehydrogenase (LDH) activity and cell viability in H9c2 cells subjected to anoxia/reoxygenation (A/R) injury. (a, b) Effects of TSN on the expression of 14-3-3*η* in H9c2 cells subjected to A/R injury by Western blot analysis. Untreated cells which served as the AD14-3-3*η* RNAi group were incubated with adenovirus containing 14-3-3*η*siRNA, and the AD-scrRNA group was incubated with empty adenovirus vector. *β*-Actin served as a loading control (a). Pretreatment with TSN increased 14-3-3*η* expression under A/R injury, which was silenced by AD14-3-3*η* RNAi (b). TSN pretreatment inhibited activity of LDH (c) and increased cell viability (d) under A/R injury, and this effect was silenced by AD14-3-3*η* RNAi. Values are presented as the mean ± SEM for five individual experiments. (A) *P* < 0.01 versus control group; (B) *P* < 0.01 versus A/R group; (C) *P* < 0.01 versus TSN + A/R group; (D) *P* < 0.05 versus A/R group; (E) *P* < 0.05 versus AD14-3-3*η*RNAi group; (F) *P* < 0.01 versus AD14-3-3*η*RNAi group.

To further investigate the protective effects of TSN, ad14-3-3*η* RNAi was used to downregulate 14-3-3*η* expression in H9c2 cells. As illustrated by Western blot analysis in Figures 2(a) and 2(b), ad14-3-3*η* RNAi prevents upregulation of 14-3-3*η* expression caused by TSN (*P* < 0.01 versus TSN); however, no effect of AD-scrRNAi was observed on 14-3-3*η* expression. Next, we determined LDH activities and cell viability (Figures 2(c) and 2(d)). Compared with the TSN group, LDH activities of the TSN plus ad14-3-3*η*RNAi group were significantly increased, and the cell viability of the TSN plus ad14-3-3*η*RNAi group was decreased (*P* < 0.01). Moreover, AD-scrRNAi had no significant effect when compared to the A/R group (*P* > 0.05). These data indicated that there was a positive correlation between the cytoprotective effect and the expression of 14-3-3*η*.

### 3.3. The Effect of Mitochondrial Translocation of Bcl-2 and 14-3-3*η* May Play an Important Role in the Protective Effect of TSN Preconditioning in H9c2 Cells Subjected to A/R Injury

**Figure 3:**
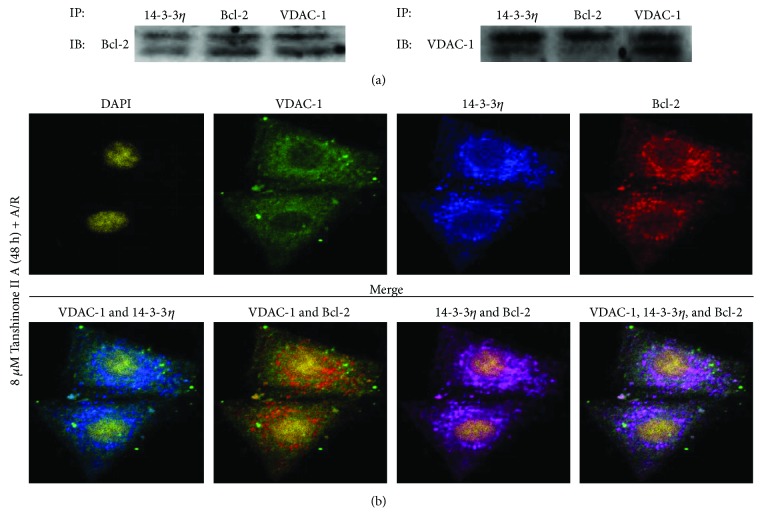
Protein interaction among Bcl-2, 14-3-3*η*, and VDAC-1 on Tanshinone IIA (TSN) preconditioning in H9c2 cells subjected to A/R injury. (a) Cells were pretreated with TSN for 2 days prior to A/R injury and extraction of proteins. Proteins were immunoprecipitated with anti-14-3-3*η*, anti-Bcl-2, and anti-VDAC-1, respectively, and immunoblotted with anti-Bcl-2 and anti-VDAC-1. (b) Cells were pretreated with TSN for 2 days prior to A/R injury. The cyan color reflects overlap of VDAC-1 and 14-3-3*η*. A yellow color reflects overlap of VDAC-1 and Bcl-2, a purple color reflects overlap of 14-3-3*η* and Bcl-2, and a white color reflects overlap of 14-3-3*η*, Bcl-2, and VDAC-1. Data were collected from three individual experiments.

**Figure 4:**
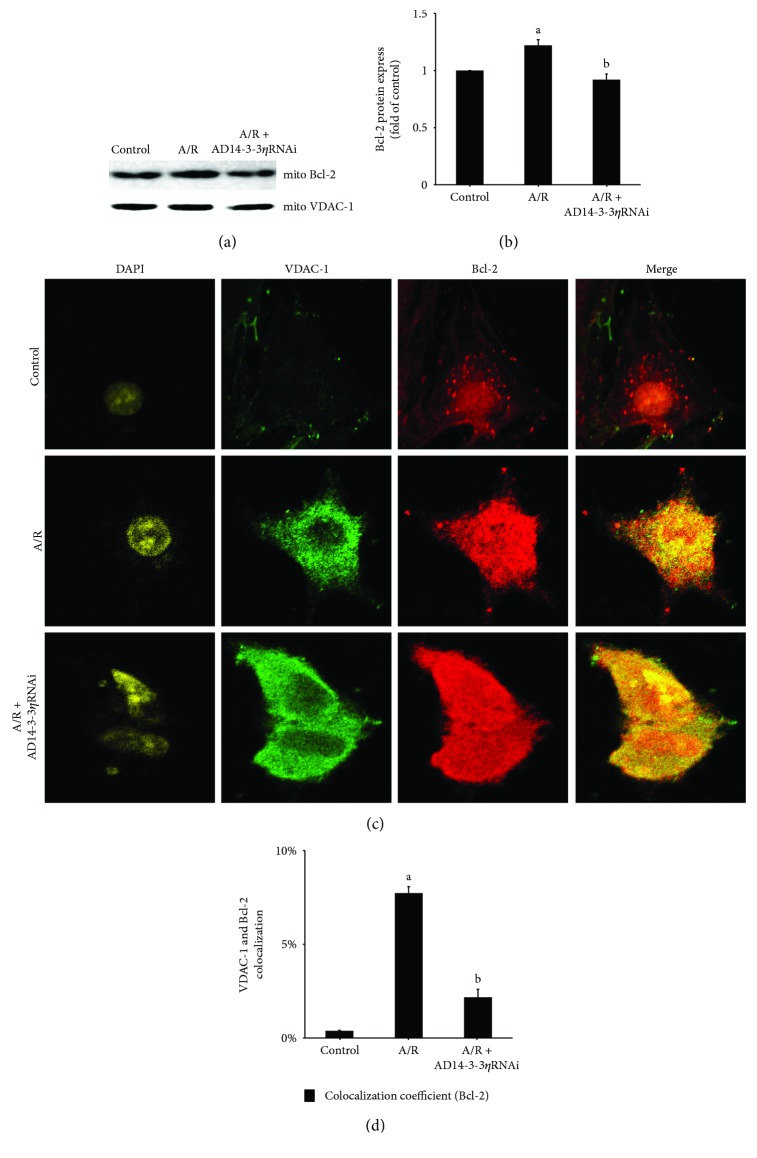
Inhibition of the translocation of Bcl-2 to the mitochondrial fraction upon 14-3-3*η* expression in H9c2 cells under anoxia/reoxygenation (A/R) injury. Mitochondrial fractions were isolated, and proteins were extracted from H9c2 cells under A/R injury. Representative Western blot results (a) and quantitative analysis (b) showing that 14-3-3*η* knockdown reduced Bcl-2 translocation from the cytoplasm to mitochondria. VDAC-1 served as a loading control. Values are presented as the mean ± SEM from three individual experiments. Representative confocal immunofluorescence images (c) or quantitative analysis (d) showing Bcl-2 translocation from the cytoplasm to mitochondria under A/R injury. This translocation was abolished by silencing 14-3-3*η* expression under A/R injury. For quantitative analysis, 10 cells were randomly selected and analyzed. Values are presented as the mean ± SEM from three individual experiments. (A) *P* < 0.01 versus control group; (B) *P* < 0.01 versus A/R group.

14-3-3*η* is a molecular partner. Our unpublished findings showed that 14-3-3*η* assisted in protein translocation. To explore the relationship among Bcl-2, 14-3-3*η*, and VDAC-1, we performed coimmunoprecipitation experiments to assess the potential interaction of TSN pretreatment 2 days before A/R treatment. In brief, proteins were immunoprecipitated with anti-14-3-3*η*, anti-Bcl-2, and anti-VDAC-1, respectively, and immunoblotted with anti-Bcl-2 first, then immunoblotted with anti-VDAC-1. The results showed that Bcl-2, 14-3-3*η*, and VDAC-1 had a noticeable interaction (Figure 3(a)). To further confirm this phenomenon, immunofluorescence assay and confocal microscopy analysis were employed. As illustrated in Figure 3(b), Bcl-2, 14-3-3*η*, and VDAC-1 colocalize after TSN pretreatment 2 days prior to A/R treatment. In addition, as shown in Figures 4(a) and 4(b), A/R injury induced Bcl-2 translocation from the cytoplasm to the mitochondria when compared with the control group. Furthermore, 14-3-3*η* knockdown reduced Bcl-2 translocation from the cytoplasm to the mitochondria when compared with the A/R group. The results were further confirmed by immunofluorescence analysis using confocal microscopy (Figures 4(c) and 4(d)). The data showed that overlap between the Bcl-2 and VDAC-1 signals was significantly increased with A/R injury and reduced when 14-3-3*η* expression was downregulated.

### 3.4. Inhibiting Mitochondrial Translocation of Bcl-2 by Silencing 14-3-3*η* Expression Abolished the Protective Effect of TSN Pretreatment on ROS Generation and Oxidative Stress in H9c2 Cells Subjected to A/R Injury

**Figure 5:**
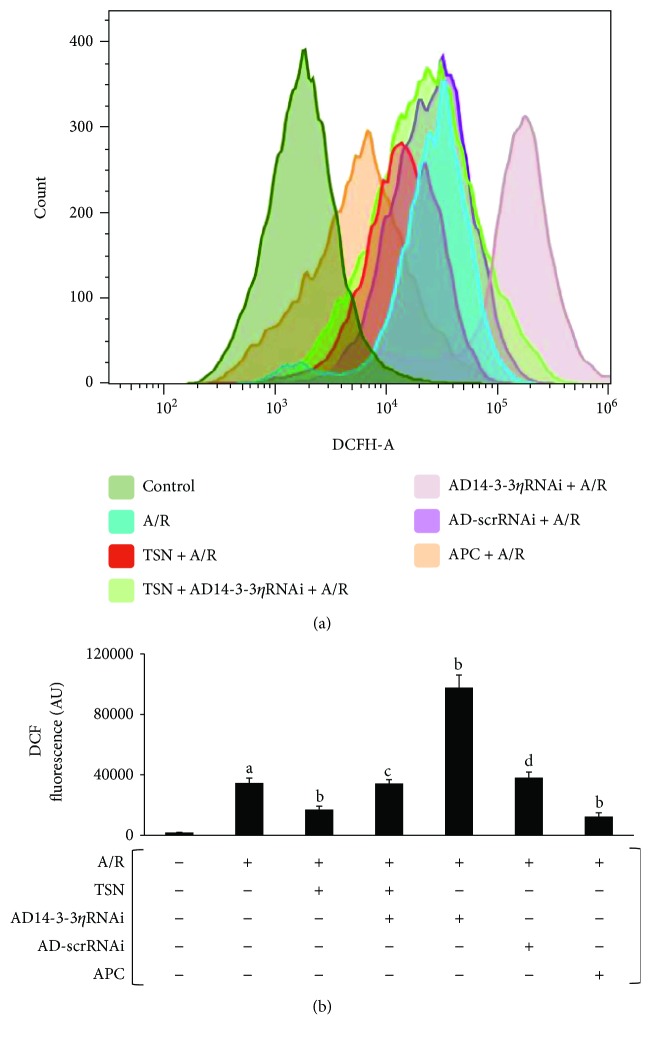
Inhibiting mitochondrial translocation of Bcl-2 by silencing 14-3-3*η* expression abolished the cardioprotective effect of Tanshinone IIA (TSN) pretreatment on the generation of reactive oxygen species (ROS) and oxidative stress in H9c2 cells subjected to anoxia/reoxygenation (A/R) injury. TSN pretreatment inhibited the generation of ROS, and this effect was reversed by AD14-3-3*η* RNAi. (a) Flow cytometric histograms of 7′-dichlorofluorescein. (b) Column bar graph of cell fluorescence for DCF. Values are presented as the mean ± SEM for five individual experiments. (A) *P* < 0.01 versus control group; (B) *P* < 0.01 versus A/R group; (C) *P* < 0.05 versus TSN + A/R group; (D) *P* < 0.01 versus AD14-3-3*η*RNAi group.

To identify the biological function of this mechanism of oxidative stress, we examined the ROS generation, the activity of antioxidant enzymes, and lipid peroxidation. ROS bursts were detected in mitochondria-mediated apoptosis. Therefore, ROS levels were used as a marker for apoptosis. As shown in Figures 5(a) and 5(b), A/R injury resulted in ROS bursts (*P* < 0.01 versus control). In addition, the TSN pretreatment and APC group showed decreased ROS production (*P* < 0.01 versus A/R group), whereas treatment with ad14-3-3*η* RNAi prevented against protection of TSN (*P* < 0.01 versus TSN group). A significant increase in the level of ROS was found in the ad14-3-3*η*RNAi-only group (*P* < 0.01 versus A/R group). Between the TSN and APC groups, no statistically significant differences were observed. It has previously been shown that oxidative stress plays an important role in A/R injury. The MDA content and SOD and GSH-Px activities are often determined to assess the level of oxidative stress. As shown in Table 1, in the A/R group, the MDA content was markedly increased (*P* < 0.01 versus control) and SOD and GSH-Px activities were significantly decreased (*P* < 0.01 versus control). In the TSN pretreatment and APC groups, a decreased MDA content and improved SOD and GSH-Px activities were observed (*P* < 0.01 versus A/R group). However, ad14-3-3*η*RNAi partly inverted the cardioprotection of TSN (*P* < 0.05 versus TSN group). No statistically significant differences were observed between the TSN and APC groups.

### 3.5. Inhibiting Mitochondrial Translocation of Bcl-2 by Silencing 14-3-3*η* Expression Abolished the Protective Effect of TSN Pretreatment on mPTP Opening and Cyt c Release in H9c2 Cells Subjected to A/R Injury

**Figure 6:**
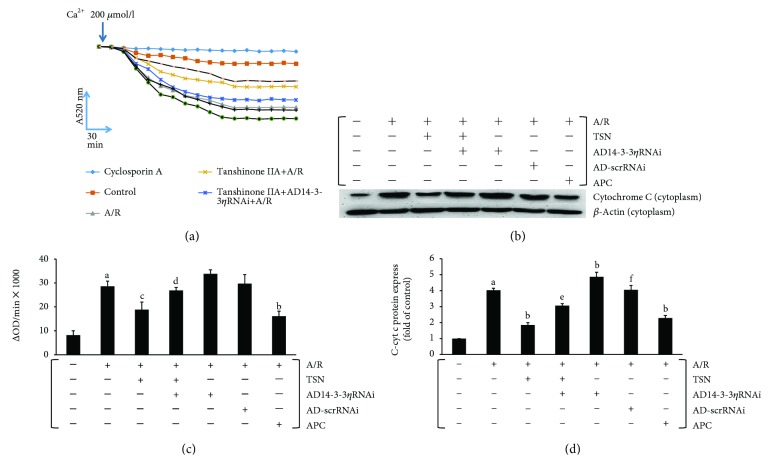
Inhibiting mitochondrial translocation of Bcl-2 by silencing 14-3-3*η* expression abolished the protective effect of Tanshinone IIA (TSN) pretreatment on mPTP opening and cyt c release in H9c2 cells subjected to anoxia/reoxygenation (A/R) injury. Ca^2+^-induced swelling of mitochondria was used to access mPTP opening. mPTP opening led to mitochondrial swelling and caused a change in absorbance at 520 nm. (a) The changes in absorbance were detected every 2 min to access the progress of mPTP opening. (b) The extent of mPTP opening was accessed by using the equation *∆*OD = *A*_520_0 min − *A*_520_20 min. (c, d) The cyt c release from mitochondria to the cytoplasm was accessed by Western blot analysis. Values are presented as the mean ± SEM for five individual experiments. (A) *P* < 0.01 versus control group; (B) *P* < 0.01 versus A/R group; (C) *P* < 0.05 versus A/R group; (D) *P* < 0.05 versus TSN + A/R group; (E) *P* < 0.01 versus TSN + A/R group; (E) *P* < 0.01 versus TSN + A/R group; (F) *P* < 0.01 versus AD14-3-3*η*RNAi group.

In mitochondria-mediated apoptosis, mPTP opening is an important factor. Therefore, we measured mPTP opening by Ca^2+^-induced mitochondrial swelling. A significant decrease in A520 absorbance was observed after A/R treatment, and TSN pretreatment receded this phenomenon (Figures 6(a) and 6(b)). Moreover, in the ad14-3-3*η*RNAi plus TSN group, the absorbance at A520 was decreased when compared with that of the TSN group. Moreover, the ad14-3-3*η*RNAi-only group showed the greatest decrease at A520. Together, these findings indicated that mPTP opening could be alleviated by treatment with TSN and that 14-3-3*η* knockdown abolished this phenomenon. Thus, 14-3-3*η* may be an important effector of TSN. No statistically significant differences were observed between the TSN and APC groups.

Upon opening of mPTP, proapoptotic factors will be released from the mitochondria into the cytoplasm and induce apoptosis. We detected the expression of cytosolic cyt c, an index of mPTP opening, by Western blotting. Figures 6(c) and 6(d) show that A/R injury induced the release of cyt c and that TSN decreased the expression of cytosolic cyt c. In addition, 14-3-3*η* knockdown reversed the effects of TSN. No statistically significant differences were observed between the TSN and APC groups.

### 3.6. Inhibiting Mitochondrial Translocation of Bcl-2 by Silencing 14-3-3*η* Expression Abolished the Protective Effect of TSN Pretreatment on Caspase-3 Activity in H9c2 Cells Subjected to A/R Injury

**Figure 7:**
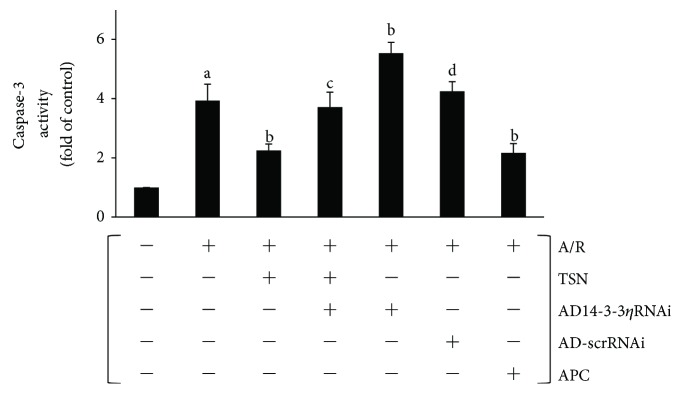
Inhibiting mitochondrial translocation of Bcl-2 by silencing 14-3-3*η* expression abolished the protective effect of Tanshinone IIA (TSN) pretreatment on caspase-3 activity in H9c2 cells subjected to anoxia/reoxygenation (A/R) injury. The cytosolic fraction was used to detect caspase-3 activity. The data shows that TSN pretreatment inhibited caspase-3 activity, and this effect was reversed by AD14-3-3*η* RNAi. Values are presented as the mean ± SEM for five individual experiments. (A) *P* < 0.01 versus control group; (B) *P* < 0.01 versus A/R group; (C) *P* < 0.05 versus TSN + A/R group; (D) *P* < 0.05 versus AD14-3-3*η*RNAi group.

The caspase-dependent pathway plays an important role in mitochondria-mediated apoptosis, and activating caspase-3-induced apoptosis is irreversible [18]. Thus, the activity of caspase-3 was measured by a colorimetric method as described previously [12]. As illustrated in Figure 7, in the A/R group, the activity of caspase-3 was markedly increased (*P* < 0.01 versus control group). Moreover, compared with the A/R group, the TSN-only pretreatment group showed a decreased activity of caspase-3 (*P* < 0.01), and ad14-3-3*η*RNAi partly abolished the protection of TSN (*P* < 0.01). Moreover, the ad14-3-3*η*RNAi-only group showed a significantly increased activity of caspase-3 (*P* < 0.01 versus A/R group). No statistically significant differences were observed between the TSN and APC groups.

### 3.7. Inhibiting Mitochondrial Translocation of Bcl-2 by Silencing 14-3-3*η* Expression Abolished the Protective Effect of TSN Pretreatment on Apoptosis in H9c2 Cells Subjected to A/R Injury

**Figure 8:**
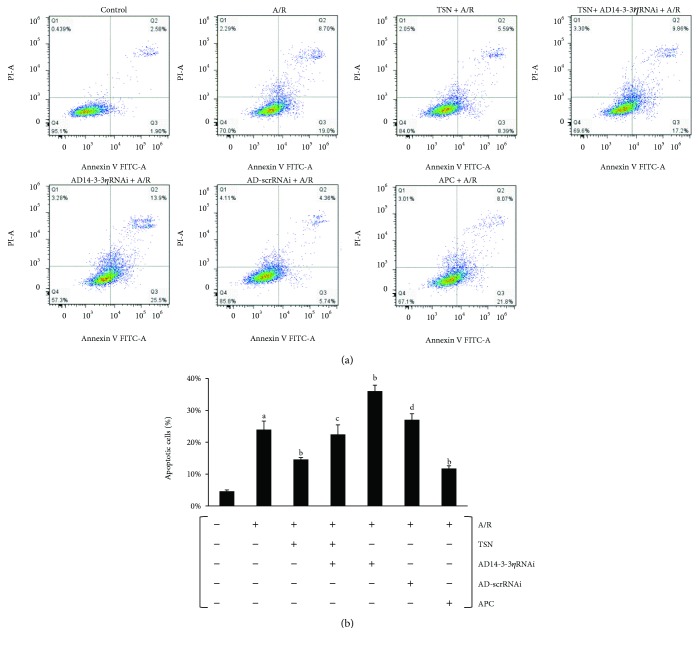
Inhibiting mitochondrial translocation of Bcl-2 by silencing 14-3-3*η* expression abolished the protective effect of Tanshinone IIA (TSN) pretreatment on apoptosis in H9c2 cells subjected to anoxia/reoxygenation (A/R) injury. (a) Representative flow cytometric dot plots (*x*-axis: Annexin V staining and *y*-axis: PI staining). (b) The apoptotic cell population was quantified and presented as a column bar graph. Values are presented as the mean ± SEM from five individual experiments. (A) *P* < 0.01 versus control group; (B) *P* < 0.01 versus A/R group; (C) *P* < 0.05 versus TSN + A/R group; (D) *P* < 0.01 versus AD14-3-3*η*RNAi group.

After A/R treatment, cells were collected and Annexin V-FITC/PI double staining and flow cytometry analysis were employed (Figures 8(a) and 8(b)). The data indicated that the percentage of apoptotic cells was increased after A/R injury (*P* < 0.01 versus control group). Moreover, treatment with TSN effectively inhibited apoptosis (*P* < 0.01 versus A/R group). However, the protective effects of TSN were reversed after infection with ad14-3-3*η*RNAi (*P* < 0.05 versus TSN group). In addition, a higher level of apoptosis was observed in the ad14-3-3*η*RNAi-only group (*P* < 0.01 versus A/R group). No statistically significant differences were observed between the TSN and APC groups.

## 4. Discussion

In a previous study, Abdukeyum et al. [10] proposed the “nutritional preconditioning (NPC)” hypothesis, which, since its introduction, has been the focus of many studies. According to the concept of NPC, the nutrients used must naturally exist, can be obtained through the diet, be nontoxic, and exhibit cardioprotective effects when used in small dosages [11]. In the present study, we showed that NPC effectually prevented myocardial reperfusion injury [11, 12]. TSN, a prime bioactive natural extractive from danshen, is considered a dietary nutrient that is used for making soup, soaking wine, and making tea, and until recently it was used as a medicine. In Asian countries, danshen has been used for hundreds of years for treating illnesses and has been commercially available in natural health stores throughout the USA and Europe [1, 19]. Dashtdar et al. reported that the extractive from danshen demonstrated lower toxicity compared to unnatural, synthetic chemical products [20]. Moreover, Hu et al. [3] reported that pretreatment with TSN for 15 days had a significant improvement on infarct size and the activity of creatine kinase in a rat model. A 4-week clinical trial showed that sulfotanshinone sodium, a sulfonate of TSN, decreased fibrinogen and improved the severity of angina pectoris in 100 unstable angina pectoris patients [21]. In our study, we showed that pretreatment of cells with 8 *μ*M TSN prior to A/R induction resulted in a time-dependent decrease in LDH activity and a time-dependent increase in cell viability. To determine the protective effects of TSN, we established an APC-delayed model that served as a positive control. The APC-delayed model represented the *in vitro* equivalent of the IPC-delayed model. Similar to the protection of the IPC-delayed model *in vivo*, the APC-delayed model is a productive protection approach against A/R injury *in vitro* [22, 23]. In this study, we demonstrated that the protective effects of TSN were no less than the protective effects of the APC-delayed model. Together, our findings indicated cardioprotective effects of TSN and the potential of TSN as a natural nutrient in nutritional preconditioning.

Although we confirmed that TSN has a protective effect, its underlying mechanism of action is still unknown. In our prior studies, we showed that TSN could upregulate the expression of 14-3-3*η* (data not shown). Therefore, we evaluated the potential relationship between 14-3-3*η* and the mechanism of the protective effect of TSN. Our findings primarily confirmed that the protective effect of TSN is dependent on the expression of 14-3-3*η* and were time-dependent (Figures 1 and 2). The expression of 14-3-3*η* was induced through TSN pretreatment during A/R injury. Silencing of the 14-3-3*η* expression by Ad14-3-3*η* RNAi aggravated A/R injury. When cells were pretreated with TSN and ad14-3-3*η* RNAi, the upregulated expression of 14-3-3*η* was partly abolished and the protective effects of TSN were also antagonized. In addition, ad14-3-3*η* RNAi silenced the expression of 14-3-3*η* by degrading mRNA. These data suggested that 14-3-3*η* was the main target of TSN and may be regulated at the transcriptional level.

It is well known that the 14-3-3 family of proteins functions through protein interactions [24]. In a previous study, it was shown that 14-3-3 family proteins appear with Bcl-2 family proteins [25, 26]. Bcl-2 family proteins play critical roles in the mitochondria-mediated apoptosis pathway [27]. Zhang et al. [28] demonstrated that the mechanism for cardioprotection by pretreatment with TSN was related to mPTP opening. In the present study, we demonstrated that mPTP opening is a critical control point in the mechanism of both mitochondrial extrinsic and intrinsic damage and that Bcl-2 family proteins could regulate VDAC-1 function, which controls most of the metabolites to adjust mPTP opening [29]. mPTP opening is related to mitochondrial matrix swelling [30], and this process is prevented by Bcl-2 [31]. Studies by Mikhailov et al. [32] and García-Sáez et al. [33] reported that Bcl-2 could bind with Bax, leading to outer mitochondrial membrane pore formation. However, when bound to VDAC-1, it leads to inhibition of mPTP opening. However, the mechanism of Bcl-2 translocation from the cytoplasm to mitochondrial binding with VDAC-1 remains unclear. In our study, we identified a significant interaction and colocalization among 14-3-3*η*, Bcl-2, and VDAC-1 by pretreatment with TSN prior to A/R injury (Figure 3). These preliminary findings confirmed our hypothesis that inhibiting mPTP opening thought cytoplasmic Bcl-2 blocking VDAC-1 directly by binding with 14-3-3*η*. To further test our hypothesis, we isolated mitochondria and extracted mitochondrial proteins to determine the expression of Bcl-2 (Figures 4(a) and 4(b)). Our data indicated that the expression of Bcl-2 on mitochondria was increased after A/R injury when compared with the control group and that this phenomenon was reversed by decreasing the expression of 14-3-3*η*. Similar results were observed in the ratio of colocalization between Bcl-2 and VDAC-1 (Figures 4(c) and 4(d)). Taken together, we were the first to find a novel intracellular molecular mechanism. When cells were exposed to A/R injury, cytoplasmic Bcl-2 translocated to mitochondria and blocked VDAC-1 through binding with 14-3-3*η*.

In several studies, it was demonstrated that cyclophilin D was a major mPTP regulator and induced mPTP opening [30]. Various proteins, such as Hsp90 [34], PPAR*α* [35, 36], p53 [37, 38], and Bcl-2 [39], have been shown to interact with cyclophilin D and thereby modulate the mPTP. In this study, although our results confirmed that Bcl-2 through interaction with 14-3-3*η*, translocated from the cytoplasm to the outer mitochondrial membrane, Bcl-2 binds with VDAC-1 and closes the mPTP. However, it may be possible that Bcl-2 could interact with cyclophilin D and modulate the mPTP, mediated by 14-3-3*η*. Of course, this possibility would need to be confirmed by a series of additional experiments.

In our previous study, we demonstrated that inhibiting the mitochondria-mediated apoptosis pathway could effectively reduce A/R injury [40] and that inhibition of mPTP opening improved cardiac function [41, 42]. Moreover, the mechanisms of TSN-induced cardioprotection were linked to its antioxidant performance [3] and Bcl-2 prevented ROS damage, cyt c release, and activation of caspase-3 [31, 43], thereby alleviating mPTP opening, inhibiting cyt c release, and preventing apoptosis. This novel mechanism, which inhibited the mitochondria-mediated apoptosis pathway, requires further study. In this study, we confirmed that A/R injury induced a ROS outburst (Figure 5), increased the MDA content, decreased the levels of SOD, decreased the GSH-Px activity (Table 1), increased the mPTP opening (Figures 6(a) and 6(b)), released cyt c (Figures 6(c) and 6(d)), activated caspase-3 (Figure 7), and induced apoptosis (Figure 8). Moreover, TSN inhibited the ROS outburst (Figure 5), decreased the MDA content, increased the activity of SOD and GSH-Px (Table 1), inhibited mPTP opening (Figures 6(a) and 6(b)), decreased cyt c release (Figures 6(c) and 6(d)), prevented caspase-3 activation (Figure 7), and prevented apoptosis (Figure 8).

## 5. Limitations of the Study

In this study, we used H9c2 myoblast cells as the study object. The results obtained using these cells may not be representative of potential effects of primary cardiomyocytes or an intact myocardium. In addition, we used the A/R model to simulate reperfusion injury in vivo/ex vivo models, which may limit the universality of the results obtained.

## 6. Conclusions

Although cardioprotective effects of TSN have been studied in cells, animals, and human clinical trials, the approach of using nutrition preconditioning has not been explored and its mechanism of action is unknown. In the present study, we revealed novel evidence that TSN has potential beneficial effects against A/R injury. We demonstrated that the mechanism of action may be associated with upregulation of 14-3-3*η*, allowing Bcl-2 to translocate to mitochondria thereby preventing mPTP opening, inhibiting ROS bursts, decreasing cyt c release, avoiding caspase-3 activation, and preventing apoptosis. Although our study has shown the potential of TSN habitual intake and a novel mechanism in an *in vitro* model, long-term molecular and physiological relevance of these findings in animals and humans is clearly warranted in future studies.

## Figures and Tables

**Table 1:** Effects of Tanshinone IIA (TSN) on oxidative stress in H9c2 cells subjected to anoxia/reoxygenation (A/R) injury.

Groups	MDA (*μ*M)	SOD (IU/I)	GSH-Px (IU/I)
Control	1.52 ± 0.12	34.62 ± 2	195.83 ± 16.06
A/R	6.3 ± 0.86^a^	11.44 ± 2.19^a^	105.16 ± 13.78^a^
TSN + A/R	3.31 ± 0.28^b^	26.17 ± 2.74^b^	160.27 ± 11.46^b^
TSN + siRNA + A/R	5.25 ± 0.49^c^	15.58 ± 2.5^c^	103.81 ± 10.41^d^
siRNA + A/R	6.93 ± 0.6	8.76 ± 1.41	62.07 ± 5.1^e^
NC + A/R	5.9 ± 0.84	11.71 ± 2.27	99.79 ± 7.76^f^
APC + A/R	3.33 ± 0.41^a^	25.83 ± 4.31^a^	159.58 ± 8.79^a^

Values are mean ± SEM, *n* = 5 per group. ^a^*P* < 0.01 versus control group; ^b^*P* < 0.01 versus A/R group; ^c^*P* < 0.01 versus TSN + A/R group; ^d^*P* < 0.05 versus TSN + A/R group; ^e^*P* < 0.05 versus A/R group; ^f^*P* < 0.05 versus siRNA group.

## Abbreviations

APC: Anoxia precondition
A/R: Anoxia/reoxygenation
BCA: Bicinchoninic acid
BSA: Bovine serum albumin
Cyt c: Cytochrome c
DAPI: 4′,6-Diamidino-2-phenylindole
DCFH-DA: Dichlorodihydrofluorescein diacetate
DMEM: Dulbecco's modified Eagle medium
DMSO: Dimethyl sulfoxide
ECL: Enhanced chemiluminescence
FITC: Fluorescein isothiocyanate
GSH-Px: Glutathione peroxidase
IPC: Ischemic preconditioning
LDH: Lactate dehydrogenase
MDA: Malondialdehyde
mPTP: Mitochondrial permeability transition pore
MTT: Methyl thiazolyl tetrazolium
NPC: Nutrition preconditioning
PBS: Phosphate-buffered saline
pNA: *p*-Nitroanilide
PPC: Pharmacological preconditioning
PVDF: Polyvinylidene fluoride
RIPA: Radioimmunoprecipitation assay
ROS: Reactive oxygen species
SDS/PAGE: Sodium dodecyl sulfate-polyacrylamide gel electrophoresis
SOD: Superoxide dismutase
TSN: Tanshinone IIA
VDAC-1: Voltage-dependent anion channel 1.
